# Origin and phylogenetic status of the local Ashanti Dwarf pig (ADP) of Ghana based on genetic analysis

**DOI:** 10.1186/s12864-017-3536-6

**Published:** 2017-02-20

**Authors:** Richard Osei-Amponsah, Benjamin M. Skinner, Dennis O. Adjei, Julien Bauer, Greger Larson, Nabeel A. Affara, Carole A. Sargent

**Affiliations:** 1 Animal Breeding and Genetics Research Group, Department of Animal Science, University of Ghana, P. O. Box LG 226, Legon-ACCRA, Ghana; 2 Mammalian Genetics Research Group, Department of Pathology, University of Cambridge, Tennis Court Rd, Cambridge, CB2 1QP UK; 3 Palaeogenomics and Bio-Archaeology Research Network, Research Laboratory for Archaeology, Dyson Perrins Building, South Parks Road, Oxford, OX1 3QY UK

**Keywords:** Local pigs, D-Loop, Gene sequencing, SNP genotyping

## Abstract

**Background:**

The Ashanti Dwarf Pig (ADP) of Ghana is an endangered pig breed with hardy and disease resistant traits. Characterisation of animal genetic resources provides relevant data for their conservation and sustainable use for food security and economic development. We investigated the origin and phylogenetic status of the local ADP of Ghana and their crosses with modern commercial breeds based on mtDNA, *MC1R*, Y-chromosome sequence polymorphisms, and genome-wide SNP genotyping.

**Results:**

The study involved 164 local pigs sampled from the three agro-ecological zones of Ghana. Analyses of the mitochondrial D-loop region and Y-chromosome sequences revealed both European and Asian genetic signatures, with differences between the geographical zones. Black coat colour is the most predominant within the breed, with black *MC1R* alleles of both Asian and European origin. European alleles for spotting are present at a low frequency in the sample set, and may account for the occurrence of spotted piglets in some APD litters. PCA analysis of SNP data revealed a strong location and breed effect on clustering of local Ghanaian pigs. On a global level, Ghanaian local pigs cluster closely with European pigs of commercial origin, but we identified intervals via F_ST_ analyses that may elucidate loci for ADP specific traits.

**Conclusions:**

The presence of both European and Asian contributions, with differences between geographical zones probably reflects trading and colonial influences. Understanding the effects of admixture on important adaptive and economic traits of the ADP and other local breeds in Africa is critical for developing sustainable conservation programmes to prevent the decline of these genetic resources.

**Electronic supplementary material:**

The online version of this article (doi:10.1186/s12864-017-3536-6) contains supplementary material, which is available to authorized users.

## Background

Pigs (*Sus scrofa)* display enormous phenotypic diversity in terms of shape, colour, size, production and reproduction abilities. Indigenous pig breeds in China, for instance, are well-known for their unique reproductive and lactation performance, good meat quality, strong adaptability and disease resistance traits [1].

The Ashanti Black Forest Dwarf Pig of Ghana, commonly called the Ashanti Dwarf Pig (ADP), is a local breed raised at the subsistence level in mixed farming systems in Ghana [2, 3]. In terms of phenotypic advantages the ADP is hardy; the breed can survive under poor management, and typically subsists by rooting and scavenging. Pigs are more resistant to heat stroke [3–5], and remain more active than introduced breeds across a range of environmental conditions. They are also considered less susceptible to the majority of local diseases and parasites [6, 7]. For other traits of economic importance, the meat of the ADP is considered superior to exotic pigs [8], it has a low demand for feed [9], and good mothering ability. The latter is particularly important in free ranging pigs where there is the need to defend piglets from predators. The ADP is also unusual in that it can digest fibrous matter and tannin-rich diets more efficiently than imported genotypes [5, 10, 11].

Despite these advantages, ADP, like other local pigs, are smaller than the imported commercial breeds [8], with low growth rates, and poorer reproductive performance. The average mature body weight is 60 kg, litter size is 5–7 piglets, and there is high (22.3%) pre-weaning mortality based on piglet deaths from all causes [12–14].

Ghana’s report on Animal Genetic Resources (AnGR) indicates that, apart from the indigenous ADP, there are various locally adapted exotic breeds as well as crosses between these exotics and the local ADP [15]. Our experience in Ghana is that some farmers who have crossed ADPs with exotic breeds have generated animals with relatively high growth rate, but their ability to forage on fibrous feed, and resistance to diseases are reduced. Thus, in spite of its relatively low cost of production, the ADP has over the years faced a threat from exotic breeds such as the Large White, Landrace and Duroc [6].

Genetic characterisation of the ADP, including an understanding of its historical origin, remains an unknown, whilst phenotypic distinction between purebred and F_1_ crossbred pigs is often difficult. Recently, Adjei et al*.* [5] reported that there is a wide variation in the morphological attributes of the local pig populations. In general, animals classified by owners as ADPs presented a concave head profile (85.9%), black coat colour (67.5%), plain coat colour pattern, erect ears (84.7%) projecting backwards (52.2%) and a short cylindrical snout. Although the majority had short and straight body hair type, others had long and dense, or long and curly coats. A few had a straight back line but the majority were swaybacked (84.7%). Other local pigs in the survey had semi-lop ears, straight head profile, white and black coat colour type, patchy and spotted coat colour patterns, and long and cylindrical snouts. It is possible these differences in phenotypes occur owing to genotypic differences in the local swine gene pool, reflecting their admixture, which could potentially dilute highly beneficial adaptive genes.

In this study, we have collected information from local producers about their stock, whether there is known to be crossbreeding with exotic breeds in the local pig population, or whether farmers believe their animals to be pure ADP. Based on the information provided, the genetic analysis allows an assessment of the accuracy of the classification of “crossbred” or “purebred” ADP, alongside investigating the potential origins and genetic diversity of the ADP and other local pig populations in Ghana. The ADP has co-evolved to adapt to its environments, and loss of animals means depletion of favourable alleles from the wider gene pool which may be equally important for the improvement of exotic breeds in the future. Addressing questions about the numbers of animals, and what genetic factors contribute to their niche traits will be a major step toward the development of sustainable conservation and improvement programmes to prevent ADP decline and extinction [16], [17].

A number of approaches have been developed to study the origin, genetic variation and unique attributes of animal genetic resources. Mitochondrial DNA (mtDNA) sequences generate phylogenetic trees at several taxonomic levels, from within species to among orders (e.g. [18, 19]). MtDNA is maternally inherited, haploid, non-recombining and its evolutionary rate of base substitution is much faster than that of nuclear DNA [20]. Thus it can be used to follow the maternal contributions within the porcine domestication process [21, 22]. In contrast, fragments of sequence from the Y chromosome have been analysed to study paternal lineages in domesticated pigs [23, 24]. These studies show that there is more than one Y chromosome lineage in domestic breeds and European wild boar based on the combinations of major alleles on the non-recombining portion of the Y chromosome (NRY). Four major Y chromosome populations can be identified based on the sex determining region Y (*SRY*) gene which is responsible for sex determination in mammals [25–27]. Mitochondrial, *SRY* and additional NRY sequence polymorphisms are described in this study as part of the analysis of the genetic origins of the ADP.

Coat colour is another important trait [28] and among the contributing genes, the melanocortin receptor 1 (*MC1R*) locus is the most consistently polymorphic [29]. Upon stimulation, the receptor regulates the balance between the two pigments eumelanin (black) and pheomelanin (red) produced by melanocytes [30]. Mutations in the *MC1R* gene affect coat colour in pigs [31, 32]; loss-of-function mutations are associated with recessive red coat colour or spotting, whereas dominant black colouring is linked with mutations affecting *MC1R* signalling [29]. As black is the dominant colour of the ADP, sequences from ADP or local cross-bred animals, were compared against the porcine reference genome and matched to haplotypes defined in [33].

Based on the sequences from selected DNA regions, results suggest the ADPs have both European and Asian ancestry, with a difference between animals from the north and south of the country. Both mitochondrial data and coat colour suggest a stronger contribution from Asian genetics in the north of Ghana. An F_ST_ analysis identified regions of the genome differing between ADPs and European commercial pigs, Chinese pigs and Duroc. Within these regions, we have identified intervals with genes for lipid metabolism, skeletal development and thyroid function which provide signatures for organoleptic qualities, lean carcass and body size and the potential for their preferential selection in the ADP.

## Results

**Fig. 1:**
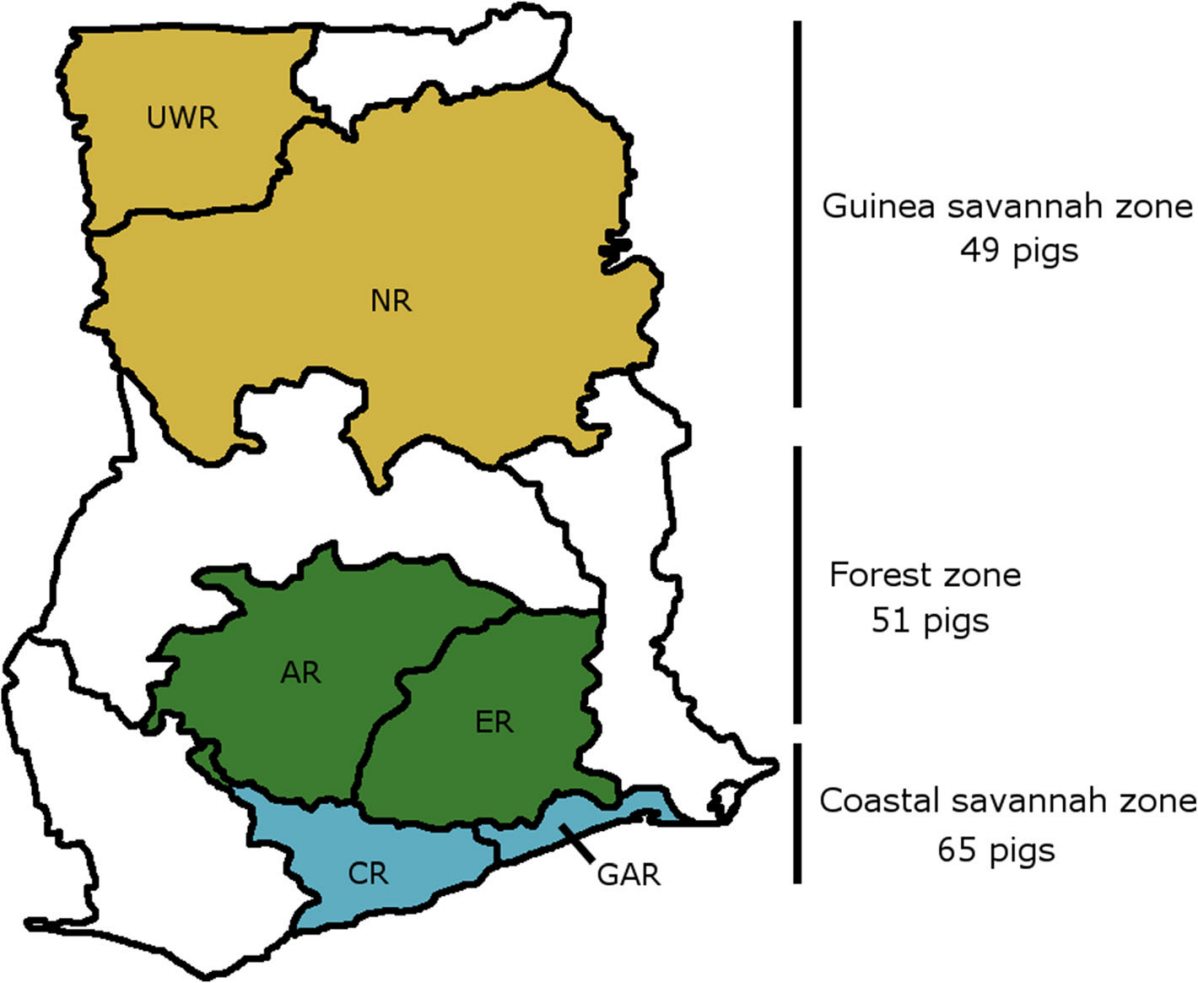
Map of Ghana The map shows the agro-ecological zones of Ghana and composite regions where pigs were sampled. The numbers of pigs per area are given on the right. (UWR = Upper West Region; NR = Northern Region; AR = Ashanti Region; ER = Eastern Region; CR = Central Region; GAR = Greater Accra Region)

For investigation of the genetic relationships, and evaluation of local Ghanaian pigs, 165 animals were sampled from the agro-ecological zones as shown in Fig. **1**. A full list of samples can be found in Additional file 1: Table S1.

### Mitochondrial haplotype analysis of sequences

**Fig. 2:**
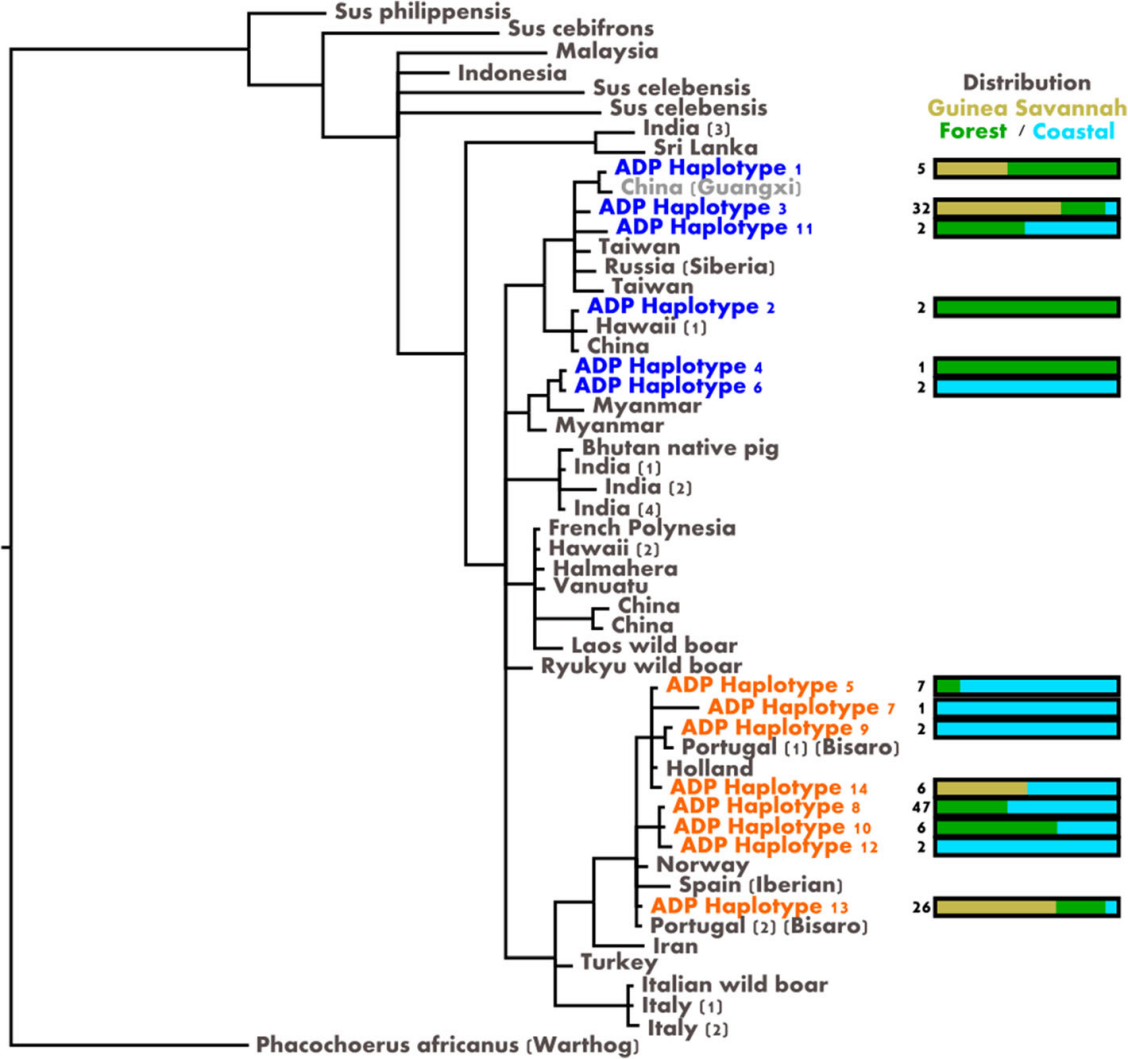
A Bayesian phylogenetic tree based on analysis of the mitochondrial D-loop region. The panel shows the local haplotypes (in *blue*) clustering with sequences of Asian origin, and those (in *orange*) clustering with the sequences of European origin. To the right of each haplotype, the number beside the bar gives the total for individuals that share the haplotype, and the bar shows the regional distribution. The African warthog was used as an outgroup

Mitochondrial DNA sequence analyses of 140 animals were used to develop a Bayesian phylogenetic tree (Fig. 2). Ghanaian local pigs clustered into 2 clades made up of 14 haplotypes, of which 8 clustered with European and 6 with Asian *Sus scrofa* haplotypes. Six of the sequences had perfect matches to existing entries in the GenBank database, whilst the remaining 8 were unique to this study (Additional file 1: Table S2). The majority of sequences from animals sampled in this study fell into three haplotypes (haplotypes 3, 8, 13: 75%). Two fall into the European clade (haplotypes 3, 13) and one into the Asian clade (haplotype 8).

#### Mitochondrial genetic distances between ADPs and other pig breeds

The clustering of haplotypes into European and Asian clades was confirmed using base substitution data, with smaller genetic distances observed between the two major European haplotypes identified in this study compared to the genetic distances between the major Asian and European haplotypes (Table 1). Globally, the mitochondrial sequences from the ADP of Ghana were closer to European than Asian sequences, as shown in Table 2.

**Table 1:** Mean genetic distances between the three predominant mtDNA haplotypes

ADP haplotype	3 (Asian)	8 (European)	13 (European)
3		*0.008*	*0.008*
8	0.026		*0.003*
13	0.026	0.004	

Three haplotypes predominate in the animals sampled (see Fig. 2). The number of base substitutions per site from averaging over sequence pairs between haplotype groups are shown. Standard error estimate(s) are shown in the upper diagonal (italicised). Haplotype 3 is within the Asian clade; haplotypes 8 and 13 are within the European clade

**Table 2:** Within breed genetic distance of various *Sus* groups from mtDNA comparisons

Breed	ADP	Asian	European	Pacific	*Sus* outgroups	African Warthog
ADP		*0.005*	*0.003*	*0.005*	*0.007*	*0.013*
Asian	0.027		*0.005*	*0.004*	*0.006*	*0.014*
European	0.018	0.027		*0.005*	*0.007*	*0.014*
Pacific	0.026	0.022	0.025		*0.006*	*0.014*
*Sus* outgroups	0.043	0.038	0.042	0.033		*0.012*
African Warthog	0.095	0.099	0.096	0.095	0.090	

The number of base substitutions per site from averaging over all sequence pairs between groups is shown. Standard error estimate(s) are shown in italics in the upper diagonal; ADP = Ashanti Dwarf pig

### SRY Sequencing and chromosome Y haplotypes

*SRY* sequences were obtained from 33 males: 21 from animals selected from the designated ADP populations and 12 from local crossbred animals. All but two animals had the *SRY* haplotype found to predominate in European animals as described in [24]. Two animals, one from the ADP population, and one from the crossbred population, both of the Ashanti region (AR), had haplotypes previously observed in Tamworth and some Asian breeds [24]. No novel *SRY* sequences were detected.

Thirty-three males (22 ADP, 2 exotic and 9 local crossbred) were identified from the genome wide genotyping panel. One individual had previously been classified as female (animal 163), but appears to be karyotypically male based on both chromosome X and chromosome Y SNP data. One individual originally assigned as male in the sample record sheet appeared to be female, based on the SNP data (animal 149). All other animals were genotyped in agreement with the recorded genders. Misclassified animals appear to be a recorder error, following cross-referencing of collection records to verify every animal included in the genotyping panel. Therefore, no animals were excluded from further analysis. Of the genotyped panel, 22 DNA samples from males were in common with those selected for *SRY* sequencing. The extended Y chromosome haplotypes were in agreement with the previously identified *SRY* haplotypes for all of these animals (Additional file 1: Table S3).

### Coat colour in Ghanaian local pigs

The ADP of Ghana, although predominantly black, displays other coat colours and patterns. These can be observed even in the offspring of selected “purebred” black-coated ADPs, suggesting the interplay of multiple coat colour genes in the phenotypic outcome (Additional file 2: Figure S3). Analysis of the collected data shows that the local animals include spotted, white or belted animals, as well as those with black coats (Additional file 3: Figure S4).

Since black coat colour is largely determined in mammals by the dominant alleles of the *MC1R* gene, the nature and origins of these alleles in Ghana were investigated through DNA sequence analysis.

### MC1R gene distribution in Ghanaian local pigs

The spectrum of coat colours exhibited in domestic animals has arisen both through the influence of human intervention, with specific colours and patterns being preferentially selected, and as a consequence of genetic bottlenecks in the breeding populations [34]. A series of alleles of the *MC1R* gene have been identified in pigs. Six major allelic variants have been reported corresponding to five different patterns of expression [31, 32]. The wild-type (*E* ^*+*^) allele, found in wild boars, corresponds to haplotypes 0101 (MC1R*1) and 0102 (MC1R*5). Analysis of black coated pigs has revealed the existence of at least two dominant mutations, which appear to have arisen independently in populations of Asian or European origin. Large Black and Black Meishan pigs carry the Asian dominant black allele (*E* ^*D1*^) corresponding to 0201 (MC1R*2: *Leu102Pro, Val95Met)*; whereas Hampshire pigs carry the European dominant black allele (*E* ^*D2*^) corresponding to 0301 (MC1R*3: *Asp124Asn*). Landrace, Yorkshire and Pietran carry the spotting allele (*E* ^*P*^) corresponding to 0501 (MC1R*6: *nt67insCC, Asp124Asn*) which causes black spotting on a red or white background [29, 33]. The black spots are attributed to the recovery of *MC1R* protein function via somatic mutations leading to restoration of the open reading frame [32]. The Duroc breed carries the red coat colour allele (*e*) corresponding to 0401 (MC1R*4: *Ala164Val* and *Ala243Thr*) [35].

In total, 86 Ghanaian pigs were fully or partially sequenced for the *MC1R* gene and promoter region. Control sequences from purebred red Duroc, and Large White animals were included and compared with the reference porcine genome.

The identifiable haplotypes for *MC1R* represented the European 301 (E^D2^; dominant black), Asian 201 (E^D1^; dominant black), European 501 (E^P^; spotting) and European 401 (red) variants of the gene. Sequence signatures for the respective promoters were confirmed by BLAST analysis of our data with *MC1R* entries in the public databases. Using these four major identifiable haplotypes, genotypes were predicted in the Ghanaian population, (Additional file 1: Table S4). For animals with incomplete or inconclusive sequence information, alleles were assigned of ‘Asian’ or ‘European’ origin based on the available data. Four animals (5, 143, 150, 158) have single base sequence deviations that do not fit the previously reported alleles. These may represent local variants of the major haplotypes, especially as the same sequence data are observed twice in animals from the Upper West Region (UWR; 150 and 158): further work with additional samples from local Ghanaian pigs is required to confirm these observations.

As with the mitochondrial data, the occurrence of Asian alleles was higher in animals from the Guinea Savannah zone than those from the other regions (Additional file 4: Figure S5). Further discussion of the distribution of alleles is given in Additional file 5.

### Genotyping analysis

Genotyping data were used to perform comparisons using PCA analyses, genome wide levels of homozygosity, and an F_ST_ comparison of the ADPs against other world pig breeds.

The PCA analysis of local Ghanaian animals is shown in Additional file 6: Figure S6A. ADPs form two main groups, based on geographical location: those from the Guinea Savannah zone are the most tightly grouped, followed by those from the Coastal zone (particularly GAR), with the Forest zone animals being more diffusely scatted across the plot. In general, the crossbred pigs tended to be closer to the exotic (European breed) animals, as might be predicted based on known introduction of European breed genetics. When data from other world pig breeds are included, the Ghanaian pigs cluster more closely with European commercial breeds than with Asian pig breeds (Additional file 6: Figure S6B).

#### F_ST_ analysis

We performed an F_ST_ analysis between two ADP subgroups and between the ADPs and European and Chinese breeds. The first ADP subgroup was selected from the Guinea Savannah zone, and formed a distinct cluster in PCA analysis (Additional file 6: Figure S6A set 1). The second subgroup came from the Coastal zone (Additional file 6: Figure S6A set 2). The two major subgroups of ADPs (Guinea Savannah and Coastal) comprise 17 animals each.

Genome averaged Fst values are consistent with the mitochondrial data: the ADPs are genetically more similar to European Commercial breeds than to either Duroc or Chinese breeds (Table 3). Interestingly, the two ADP subsets are as distant to each other as to the European breeds.

**Table 3:** Genome average F_ST_ values for each of the two ADP populations

	Guinea savannah ADPs	Coastal ADPs
Coastal ADPs	0.131	
European	0.085	0.105
Chinese	0.334	0.319
Duroc	0.196	0.191

The two populations from the Guinea Savannah zone and coastal zone were compared against European commercial breeds, Duroc and Chinese breeds. Values are based on the genome wide average. Both sets are as distant from each other as from the European commercial breeds, based on this analysis

Comparison of ADPs against European commercial breeds may help to identify regions that distinguish these local pigs from their most similar relatives. Forty-two genomic intervals contain SNPs with high F_ST_ (SNP F_ST_ in 99^th^ percentile, within a 100 kb window with average F_ST_ greater than the 95^th^ percentile: for an example see Additional file 7: Figure S8) between both ADP subgroups compared independently against the European commercial breeds. The intervals and gene content are summarised in Additional file 1: Table S5, which also indicates regions of difference against Chinese, Duroc, and between ADP groups. Notably, several of the genes in the regions distinguishing ADPs from European breeds relate to skeletal morphology and body size; for example, the transcription factor ligand dependent nuclear receptor corepressor like (*LCORL,* see Additional file 8: Figure S7), the enzyme inositol monophosphatase domain containing 1 (*IMPAD1*)*,* and calcium channel regulator stromal interaction molecule 2 (*STIM2*). A further two regions have genes related to melanin production; the KIT proto-oncogene receptor tyrosine kinase, which regulates melanocyte migration (*KIT*)*,* and the transient receptor potential cation channel subfamily M member 1 (*TRPM1*), thought to be involved in melanin production (see [36] for a discussion of genes involved in coat colour variation).

Few significant categories of genes clusters were identified using DAVID gene enrichment analysis against a human gene background to maximise information retrieval [37, 38], although genes identified at the peak SNP of F_ST_ regions in both ADP sets against other pig populations (Additional file 1: Table S5) can be grouped according to human gene-disease associations (Additional file 1: Table S6 & S7). When all loci under the F_ST_ peaks are included in the analyses, genes in behavioural classes show significance (p < 0.5) in the comparisons against all but the Chinese breeds. The two ADP groups show differences between behavioural, cardiovascular, metabolic and haematological functional classes. When only genes containing peak SNPs are classified, although values are not significant owing to the small numbers of loci analysed, ADPs still differ from European and Chinese breeds in disease classes related to cardiac function. ADP comparisons with Duroc also identify categories related to body weight and metabolic function. Individual loci containing peak SNPs analysed for disease associations are given in Additional file 1: Table S7.

## Discussion

The Ashanti Dwarf Pig (ADP) has often been held as an example of a local pig breed with important genetic characteristics worthy of preservation and conservation. To preserve and enhance the breed, the Babile Pig Breeding Station was established in 1995 by the Ministry of Food and Agriculture in a National Livestock Services Project (NLSP). Through selective breeding, it has worked to generate ADP breeding stock with superior reproductive traits, both for local farmers and NGOs who use livestock as a medium in their poverty alleviation strategies. However, the population of ADP at the station has been reducing (from 382 in 2011, to 219 in 2015) due to lack of funds. This poses a serious threat to these valuable genetic resources and calls for urgent intervention. In this study, we further show that the animals described by local Ghanaian farmers as ADP may in fact derive from pigs of different ancestries. This raises the question of what is an ADP; if it is a distinct breed, then our results indicate that the pure Ashanti pigs themselves may exist in far fewer numbers in Ghana than previously thought.

The history of pigs in Sub-Saharan Africa is unclear. The importation of European breeding stock both during and post the colonial period in Africa has diluted the ancestral genetic signatures of local animals [39]. Similarly, a clear genetic dichotomy between East and West African local pigs has been reported based on Y, mitochondrial and autosomal data [40]. This found that local pig haplotypes from West Africa (Nigeria and Benin) clustered together with European haplotypes with a mean frequency of 50% and, to a lower extent, with Near Eastern *Sus scrofa* populations. More significantly, pigs from West Africa did not display any of the haplotypes that are characteristic of Asian (Far Eastern) populations. Conversely, DNA samples from pigs of East Africa demonstrated the presence of Asian alleles. This might be as the result of direct introgression of local animals with Far Eastern breeds, or introduced through a European intermediary, given that British breeds were strongly admixed with Chinese pigs in the 18–19th centuries [40, 41].

### Mitochondrial sequences

Analysis of SNP polymorphisms in the D-loop sequence of mtDNA has been used to describe variation in putative wild ancestor and modern domestic pig populations [42–44]. In other studies, significant differentiation between European and Chinese domestic pigs has also been revealed by mtDNA analyses [21, 45–47]. The presence of both European and Asian MtDNA haplotypes in all six regions sampled indicates an admixed local pig population in Ghana. However, there is a distinct gradient with Asian sequences more common in the Guinea Savannah Zone, and European sequences on the coast (Additional file 9: Figure S2). Here, the predominance of European mtDNA haplotypes may be explained by the influence of Europe on Ghana as a result of colonization and imports of European breeds by the Ghanaian government in the recent past to boost pig production.

### Y chromosome analysis

Results of the present study on the paternal ancestry of local pigs in Ghana show that almost all the tested samples had Y chromosome signatures commonly found in multiple European breeds, with only two animals having *SRY* sequence data comparable to the Tamworth. The extended Y haplotype signature observed in Tamworth was confirmed in the single male genotyped on the Illumina panel. However, such Y chromosome data cannot rule out non-European origins or admixture, since the observed Y lineages are also found in Asian pig breeds [24].

### Coat colour and *MC1R*

Unlike Chinese domestic pigs which displayed low diversity of *MC1R* [48], Ghanaian local pigs show considerable variation in this gene. This indicates either acquired mutations [31] or an admixed population. Interestingly, the distribution of Asian and European dominant black variants broadly mirrors that of the mitochondrial data: ADP samples from the Guinea Savannah show a higher percentage of the Asian allele (almost 70% of all *MC1R* variants), and ADP samples from the coast a higher percentage of the European allele (almost 40% of all variants). The *E* ^*P*^ allele is also common in the local Ghanaian population, mainly in crossbred animals, but also in those classified as ADPs. In general, local crossbred pigs carrying at least one *E* ^*P*^ allele had white or spotted coats, whilst crossbred pigs of other genotypes were black, belted and white. The presence of white coated crossbred animals genotyped here as homozygous for dominant black alleles is also indicative of the influence of epistasis between coat colour loci, for example the dominant white *KIT* allele, in the local population.

### Genome-wide analysis

The Illumina Porcine SNP60k BeadChip [23] has been used to identify SNP associated with, for example, reproduction traits in the Finnish Landrace pig breed to provide valuable candidates for possible marker-assisted selection [49]. It has provided a genome wide overview of “indigenous” local pig populations [50] and been used to determine population structure, linkage disequilibrium (LD) pattern and selection signature in Chinese and Western pigs [51]. In this study, we have used the same chip to investigate the polymorphisms present in the local Ghanaian pig populations. The principal component analysis (PCA) of the local pig populations revealed distinct clustering based on the origin of the samples from across the three agro-economical zones (Additional file 6: Figure S6). Based on biological knowledge, PCA1 probably represents geographical distribution, and PCA2 genetic distance. At the whole genome level, the ADP sampled from Greater Accra may be genetically similar to animals from the Guinea Savannah zone. Crossbred animals are generally closer to the exotics (European commercial breeds), and animals nominally called ADPs from the forest zone (AR and ER) and the CR seem to be the most diverse. In the PCA analysis that used data from breeds collected from Europe and China, all Ghanaian local pigs, irrespective of classification, overlapped and clustered with the commercial European breeds represented by the inclusion of data from Large White and Landrace samples.

### F_ST_ data reveal genes that may underlie the phenotypic distinctiveness of ADPs

The F_ST_ analysis reveals regions of the genome that have greater or lesser genetic similarity between populations. The regions we identified distinguishing ADPs from European commercial breeds contain intervals with genes known to relate to body size and shape, previously reported as genomic intervals undergoing selection in pigs and other mammals, such as *LCORL* and the pleomorphic adenoma gene 1 *PLAG1* [52, 53]. The LCORL protein is a transcription factor implicated in humans in effects on skeletal size and adult height [54]; PLAG1 is developmentally regulated, often associated with salivary gland neoplasms, and has been linked with growth rates in cattle [55]. These genes may help explain the short stature of the ADPs compared to other breeds, and will be useful targets for more detailed study.

We propose that pigs from the southern part of Ghana should be more admixed due to colonisation, and relatively more activity in terms of trade and movement of people and animals to that part of the country. The F_ST_ data are in agreement, suggesting the ADP samples selected from the extremes of Ghana are as far distant from each other as each subgroup is from European commercial breeds. Correspondingly, pigs from these regions also show more variation in mitochondrial DNA and *MC1R* sequences. Animals from the Northern part of the country have higher percentages of Asian signature sequences at the selected loci, but it is currently unclear if this is indicative of historic breeding bottlenecks or points to two distinct geographical origins of the animals currently classified as Ashanti Dwarf Black pigs.

## Conclusions

Local ADPs of Ghana display genetic signatures indicative of both European and Asian origins at the loci described here. Although the ADP is nominally a black coated breed, the recent occurrence of spotted piglets in APD litters may be due to epistatic interactions, or a low frequency of the recessive *E* ^*P*^ allele in the selected populations introduced through unrecorded crossbreeding with other local pigs. The data presented suggest that morphology alone cannot be used to adequately characterise Ghanaian local pigs. It will be necessary to sample a larger population of local pigs in Ghana to find out how the adaptive and economic traits of the ADP have been affected by crossbreeding, and define allelic variants of value to the longer term animal breeding programme in Ghana.

## Methods

### Samples and DNA extraction

A total of 165 pigs made up of local ADPs, crossbreds and exotic pigs were sampled from six regions in the three agro-ecological zones of Ghana, namely the coastal savannah, forest and guinea savannah zones (Fig. **1**) between August 2013 and October 2013. Further details on the climate of each zone can be found in Additional file 5.

The samples were obtained from a total of 54 local pig farmers and 6 institutional pig farms. Further details are in Additional file 1: Table S1. The farmers/managers were interviewed to obtain information on the husbandry practices, such as their experience in keeping the local breed, and if they have ever crossbred their local stock with exotics. Ear tissues of sampled pigs were obtained using an ear notcher (Additional file 10: Figure S1) with assistance from animal production officers, veterinary technicians and extension agents of the Ministry of Food and Agriculture (MOFA). The samples were stored on field in RNAlater (tissue collection stabilization) solution (*Ambion*, USA) and later transported to the Biotechnology Laboratory of the School of Agriculture, University of Ghana, Legon. Genomic DNA was extracted from the ear tissues using the QIAGEN DNeasy Blood and Tissue Kit following the Manufacturers’ protocol after which the quality of the DNA obtained was tested using a spectrophotometer and stored at −80 °C. The DNA samples were subsequently transported to the laboratory of the Mammalian Genetics Group of the Department of Pathology, University of Cambridge for sequencing and genotyping.

### DNA amplification and sequencing

#### MtDNA sequencing

A segment of the D-loop region (approximately 680 bp) of the mtDNA was amplified from 140 local animals (81 classified as ADP and 59 classified as cross-bred) using the following primers pairs: L15387 (5′-CTCCGCCATCAGCACCCAAAG-3′ forward) and H124n (5′-ATRGCTGAGTCYAAGCATCC-3′ reverse) [18]. PCRs were set up using the manufacturer’s recommended conditions with Qiagen HotStarTaq® DNA polymerase in the presence of buffer Q. All reactions were carried out in 20 μL volumes, with 0.5 μM primers, and 20–50 ng DNA. The touchdown PCR was set at 95 °C for 15 min for the initial denaturation and *Taq* activation. This was then followed by 35 cycles at 94 °C for 1 min to denature the template DNA, 1 min at the annealing temperature and another 1 min at 72 °C The annealing temperature started at 62 °C, decreasing by one degree per cycle until 53 °C was reached. The remaining cycles were completed at an annealing temperature of 53 °C. The samples were held at 72 °C for 10 min and chilled at 4 °C until removed from the PCR machine.

PCR products were purified following agarose gel electrophoresis using ExoSAP-IT (USB Corporation, USA) following the manufacturer’s recommendations Amplicons were sequenced using Big Dye version 3.1 (Applied Biosystems). The sequencing program consisted of 30 cycles of: 96 °C for 10 s, 55 °C for 5 s and 60 °C for 4 min. The products were then run on an ABI 3100 capillary sequencer at the sequencing facility in the Department of Biochemistry, University of Cambridge. Traces were edited using Chromas version 2.2 (Technelysium Pty Ltd) before comparing in Sequencer (Genecodes Corporation). Sequences from different animals were also viewed using the MultAlin program (http://multalin.toulouse.inra.fr/multalin/), and within the ClustalW2 program (http://www.ebi.ac.uk/Tools/clustalw2/). Edited consensus sequences and polymorphisms associated with this study are deposited in GenBank under accession numbers KU306949-KU306962.

#### MC1R sequencing

Two primer pairs were used to amplify the majority of the single exon *MC1R* gene. The first pair was MF1 (5′ -GTGCGGCGGCTCTGGGCTCCAA forward) and MR1 (5′ -CCCCCACTCCCCATGCCTCCTG reverse) whilst the second primer pair was MF1 (5′ –GTGCGGCGGCTCTGGGCTCCAA forward) and MR2 (5′ –ACACCATGGAGCCGCAGATGAGC reverse). PCRs were carried out in a DNA thermal cycler [Perkin Elmer (Norwalk, CT) 9600] in a total volume of 20 μl containing 25 ng genomic DNA, 1.0 Mm MgCl_2_, 50 Mm KCl, 10 Mm Tris–HCl, pH 8.3, 200 μM dNTPs, 0.5 units Ampli-Taq Gold [Perkin Elmer (Norwalk, CT) 9600], and 0.5 μM each of forward and reverse primer. To activate AmpliTaq Gold, initial heat denaturation was carried out at 94 °C for 10 min followed by 32 cycles each consisting of 45 s at 94 °C, 45 s at 53 °C and 45 s at 72 °C. The final extension lasted for 7 min at 72 °C. Sequencing reactions were purified, run and analysed as above.

#### SRY sequencing

Primers capturing the entire open reading frame of the *SRY* gene were used to amplify DNA products from male pigs as previously described [24]. Samples were purified, run and analysed as above.

### Analysis of mtDNA sequence data

Sequences from the Ghanaian samples were trimmed to remove the amplification primer sequences, then aligned against each other to define 14 unique haplotypes. Reference sequences for each of the haplotypes were taken through the analyses defined below.

Sequence comparisons of the D-loop mtDNA were performed for indigenous Ghanaian pigs and selected porcine mtDNA sequences from the GenBank. The corresponding sequence of the African Warthog (*Phacochoerus aethiopicus)* (GenBank: AB046876) was used as outgroup. Sequences were aligned using Muscle. Evolutionary analyses were conducted in MEGA6 [56]. Genetic distances within and between breeds were calculated as the number of base substitutions per site from averaging over all sequence pairs between groups. Standard error estimate(s) were obtained by a bootstrap procedure (500 replicates). Analyses were conducted using the Tamura-Nei model [57]. The rate variation among sites was modelled with a gamma distribution (shape parameter = 0.33). The analysis involved 53 nucleotide sequences. All ambiguous positions were removed for each sequence pair. There were a total of 573 positions in the final dataset.

A Bayesian phylogenetic tree was constructed from the aligned sequences. The analysis was performed in MrBayes (http://mrbayes.sourceforge.net/index.php), using five million generations with sampling every 5000 generations. Traces were checked in Tracer (http://tree.bio.ed.ac.uk/software/tracer/) and burn-in generation was set at 1000. The final tree was visualised and annotated using FigTree (http://tree.bio.ed.ac.uk/software/figtree/).

### SNP genotyping and quality control

In this study, genomic DNA of 71 animals were genotyped using the Illumina PorcineSNP60 BeadChip following to the manufacturer’s protocol. One animal was genotypes twice as an internal control. Raw data were visualized and analyzed with the Genome Studio software (Illumina, San Diego, CA, USA).

The SNP genotype calls were exported and loaded in PLINK (Purcell et al*.* [59]) to perform the PCA analysis. 61565 SNPs were present at the start of the analysis. The filtering parameters were as follows: the maximum missing rate per SNP was set at 10%, minimum allele frequency at 5%, and maximum individual missing rate at 10%. 3305 variants were removed due to missing genotyping data, 6951 due to minor allele frequency threshold, and 4 samples due to individual missing SNP rate. After filtering the 51309 SNPs remained, with 67 individuals successfully typed.

The present raw dataset was also merged with data from a previous study using pig breeds selected from the Americas, Europe and Asia [50]. For this study, based on potential origins of local pigs, historic and current trading routes, a subset of European commercial, Iberian, European wild boar and Asian populations was selected for integration. The two sets were merged resulting in a starting set of 240 individuals with 45673 SNPs in common. The same filters were applied as above, excluding 3 individuals and 1445 SNPs, and leaving 237 individual and 44228 SNPs for the PCA analysis.

Based on the PCA analysis, subsets of tightly grouping ADP populations from the Guinea Savannah and Coastal zones were selected for further characterisation. Each of the two subpopulations comprised 17 animals.

### F_ST_ analysis

Comparisons of Wright’s **F**_**ST**_ s (as in [58]) for animals from different geographic regions was performed within PLINK v1.9b [59] with the 45673 SNPs identified above. The sex chromosome markers, and SNPs which were fixed in any of the populations under comparison were removed. In total, 39848 markers remained for comparison of each of the ADP subgroups against the Chinese and European breeds, and 32793 remained when comparing the two ADP subgroups to each other. The resulting **F**_**ST**_ data was analysed in R. The raw **F**_**ST**_ values were smoothed using 100 kb windows centred on each SNP in chromosomal order, and regions with high **F**_**ST**_ were identified. The threshold to consider a SNP as being of interest were: the individual SNP **F**_**ST**_ was greater than 99% of all **F**_ST_s, and the smoothed **F**_**ST**_ was greater than 95% of all **F**_ST_s. An example graph of smoothed F_ST_s along chromosome 7 between ADP subgroup 1 and Duroc is shown in Additional file 7: Figure S8.

Intervals identified by the analysis were scrutinised for gene content in Ensembl. The comparative genomics options in the web interface allowed identification of any transcripts not fully annotated in the current pig genomic build (Sscrofa10.2) by comparison with human (GRCh38p7). Gene lists created from these intervals of interest are shown in Additional file 1: Table S5, and were further analysed in DAVID v6.8 (https://david.ncifcrf.gov/) [37, 38]. Owing to the poorer levels of functional and disease association data in swine, all gene lists were run against a human background in order to extract the maximum information content. Significant results (*p* < 0.05) from the DAVID analysis are shown in Additional file 1: Table S6, with the peak genes and disease classifications based on human data in Additional file 1: Table S7.

## Additional files

Additional file 1: Table S1. Number of animals sampled per agro-ecological region, based on farms and locations. **Table S2**. A summary of mitochondrial haplotypes. **Table S3**. Results from Y chromosome derived polymorphisms. **Table S4**. Summary of coat colour information, sequence polymorphisms, and predicted haplotypes in the MC1R gene from animals collected in Ghana. **Table S5**. A summary of the intervals defined by FST analysis, with genes, flanking genes for each intergenic region, and genes at peaks commonly seen between between ADP subset 1 and ADP subset 2 comparisons with other breeds identified in bold. **Table S6**. DAVID functional annotation results showing clustering of genes (p < 0.5) from the intervals identified in comparisons with human alignments. **Table S7**. Information on an individual gene basis for genes containing a peak SNP, including Entrez gene summary, gene associations, and gene association classifications based on DAVID analysis with human based databases for extraction of maximum information. (XLSX 78 kb)
Additional file 2: Figure S3. Variation in coat colours of local Ashanti Dwarf pigs (ADP) of Ghana. Image A and B show spotting and patchy coat colours appearing in the litters of selected “purebred” ADPs, whilst C and D show spotted and belted patterns in local livestock of known mixed ancestry. (TIF 535 kb)
Additional file 3: Figure S4. Distribution of coat colour in local Ghanaian pigs. Panel 4A shows the distribution of coat colour in local ADP compared with local crossbred animals. Panel 4B shows the distribution of coat colour phenotypes by agro-ecological zones: Guinea Savannah (GSZ: UWR and NR); Forest (FZ: ER and AR); Coastal (CZ: CR and GAR); and by local pig classification into ADP or crossbred. In each panel, n = total number of animals sampled. (TIF 98 kb)
Additional file 4: Figure S5. Distribution of Asian and European *MC1R* alleles in local pigs. Panels A and B show the distribution based on whether the alleles defined are of European or Asian origin by local classification (ADP or crossbred) and by agro-ecological zone (GSZ = Guinea Savannah zone; FZ = Forest Zone; CZ = Coastal Zone). In panels C and D the haplotypes are defined in greater detail. In each panel, n = total number of animals sampled (TIF 158 kb)
Additional file 5: Supplementary information on sampling, methodology and analyses. (DOCX 28 kb)
Additional file 6: Figure S6. A: Principal Component Analysis of local pigs of Ghana based on SNP genotyping. (AR = Ashanti region; CR = Central region; ER = Eastern region; GAR = Greater Accra region; NR = Northern region; UWR = Upper West region). B: PCA analysis of Ghanaian pigs and European and Asian populations. (TIF 244 kb)
Additional file 7: Figure S8. F_ST_ values along chromosome 7, comparing ADP subgroup 1 and Duroc. The horizontal lines show the 99% and 95% genome-wide F_ST_ threshold values. Distinct regions of interest can be seen at ~30 Mb and 55 Mb with high F_ST_. (TIF 139 kb)
Additional file 8: Figure S7. F_ST_ values around the LCORL region of porcine chromosome 8. The upper panel shows population 1 against other European breeds, and the lower panel the population 2 ADPs against the same European breeds. The yellow bar represents the region 5’ to the *LCORL* locus, which harbours the peak SNP adjacent to this gene (*). (TIF 67 kb)
Additional file 9: Figure S2. Distribution of mitochondrial sequences across agro-ecological zones. Panel 2A shows the distribution of sequences according to their clustering into Asian or European clades by region: Guinea Savannah Zone (GSZ: UWR and NR); Forest Zone (FZ: ER and AR); Coastal Zone (CZ: CR and GAR). Panel 2B further subdivides the data according to local pig classification into ADP or crossbred. Panels 2C and 2D provide the same breakdown by sequence haplotype. In each panel, n = total number of animals sampled. (TIF 150 kb)
Additional file 10: Figure S1. Tissue sampling. Sampling porcine ear tissues using an ear notcher (A). B and C represent the procedure, the tube in D contains tissue in preservative. For each animal additional husbandry and morphological information (e.g. weight, height, length: D) was recorded on farm. (TIF 400 kb)

## Abbreviations

ADP: Ashanti Dwarf pigs
F_ST_: Fixation index (F-statistic)
MC1R: Melanocortin receptor 1
mtDNA: Mitochondrial DNA
PCA: Principle components analysis
SNP: Single nucleotide polymorphism
SRY: Sex determining region Y
