# Supplementary figures and images for: Origin and phylogenetic status of the local Ashanti Dwarf pig (ADP) of Ghana based on genetic analysis
Source: BMC Genomics. 2017 Feb 20;18:193. doi: 10.1186/s12864-017-3536-6 (PMC5319064; doi:10.1186/s12864-017-3536-6)

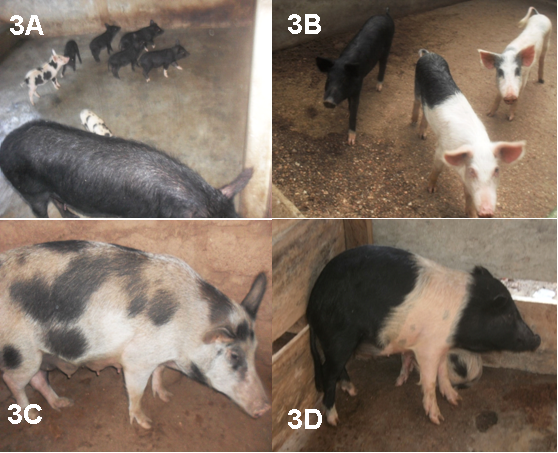

Supplement: Additional file 2: Figure S3. — Variation in coat colours of local Ashanti Dwarf pigs (ADP) of Ghana. Image A and B show spotting and patchy coat colours appearing in the litters of selected “purebred” ADPs, whilst C and D show spotted and belted patterns in local livestock of known mixed ancestry. (TIF 535 kb) [file 12864_2017_3536_MOESM2_ESM.tif]

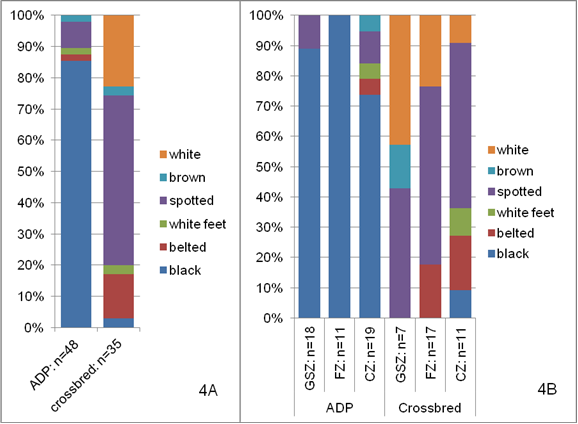

Supplement: Additional file 3: Figure S4. — Distribution of coat colour in local Ghanaian pigs. Panel 4A shows the distribution of coat colour in local ADP compared with local crossbred animals. Panel 4B shows the distribution of coat colour phenotypes by agro-ecological zones: Guinea Savannah (GSZ: UWR and NR); Forest (FZ: ER and AR); Coastal (CZ: CR and GAR); and by local pig classification into ADP or crossbred. In each panel, n = total number of animals sampled. (TIF 98 kb) [file 12864_2017_3536_MOESM3_ESM.tif]

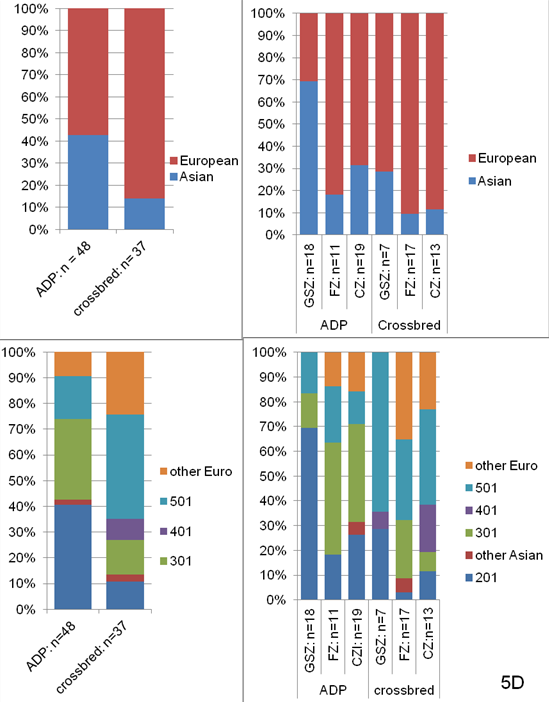

Supplement: Additional file 4: Figure S5. — Distribution of Asian and European MC1R alleles in local pigs. Panels A and B show the distribution based on whether the alleles defined are of European or Asian origin by local classification (ADP or crossbred) and by agro-ecological zone (GSZ = Guinea Savannah zone; FZ = Forest Zone; CZ = Coastal Zone). In panels C and D the haplotypes are defined in greater detail. In each panel, n = total number of animals sampled (TIF 158 kb) [file 12864_2017_3536_MOESM4_ESM.tif]

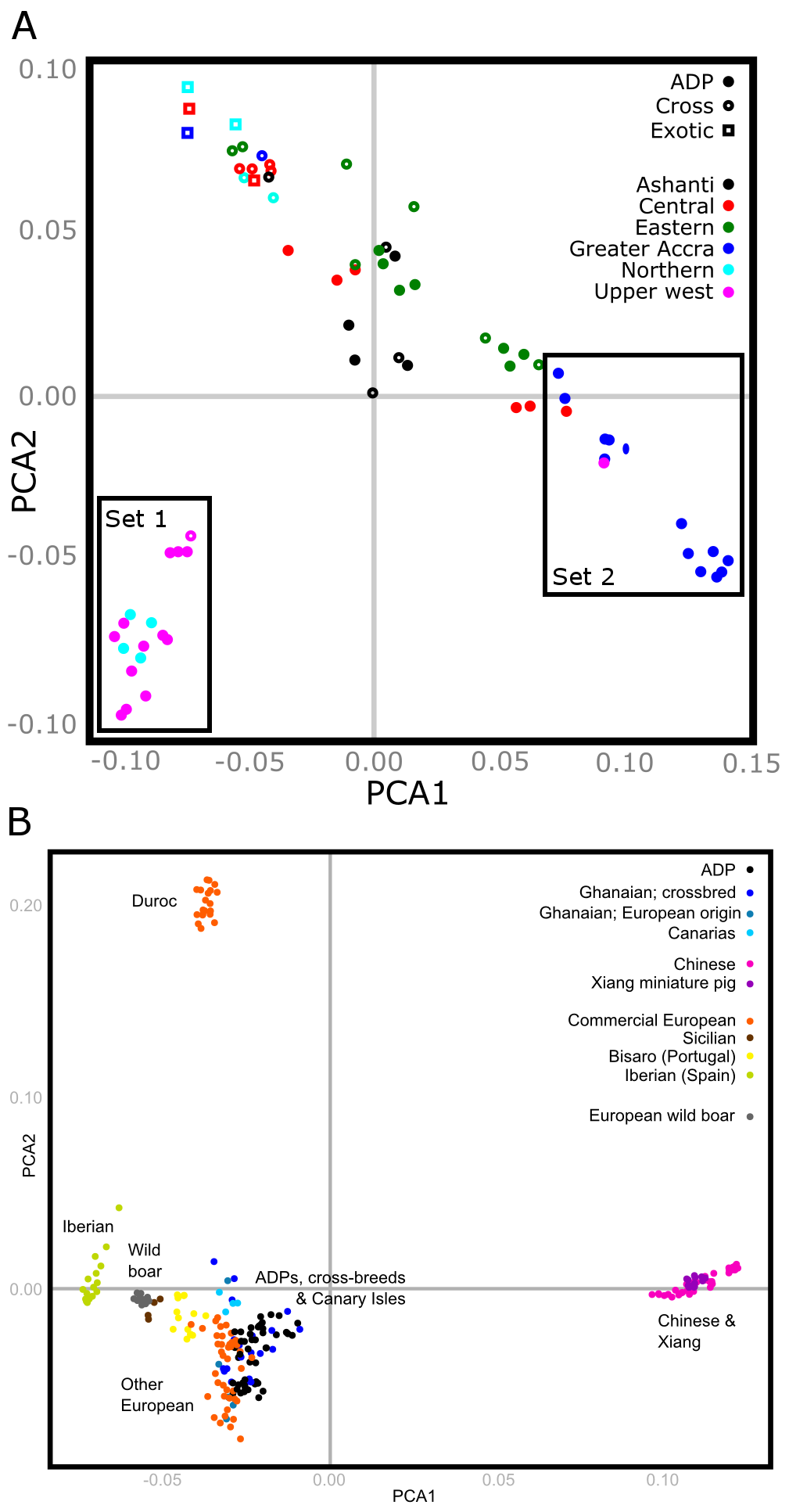

Supplement: Additional file 6: Figure S6. — A: Principal Component Analysis of local pigs of Ghana based on SNP genotyping. (AR = Ashanti region; CR = Central region; ER = Eastern region; GAR = Greater Accra region; NR = Northern region; UWR = Upper West region). B: PCA analysis of Ghanaian pigs and European and Asian populations. (TIF 244 kb) [file 12864_2017_3536_MOESM6_ESM.tif]

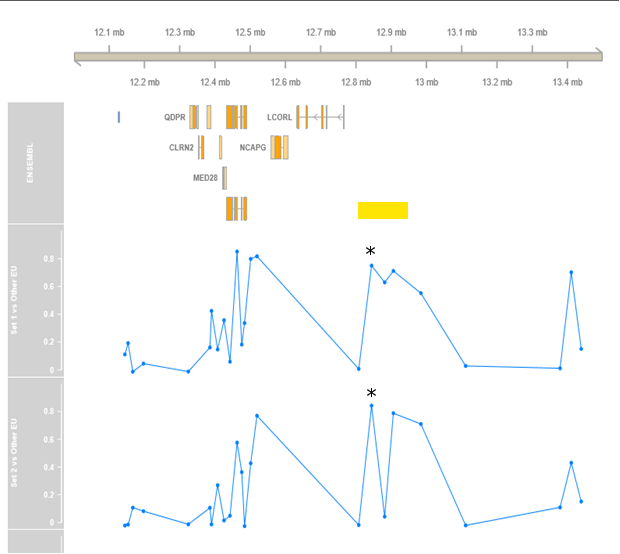

Supplement: Additional file 7: Figure S8. — FST values along chromosome 7, comparing ADP subgroup 1 and Duroc. The horizontal lines show the 99% and 95% genome-wide FST threshold values. Distinct regions of interest can be seen at ~30 Mb and 55 Mb with high FST. (TIF 139 kb) [file 12864_2017_3536_MOESM7_ESM.tif]

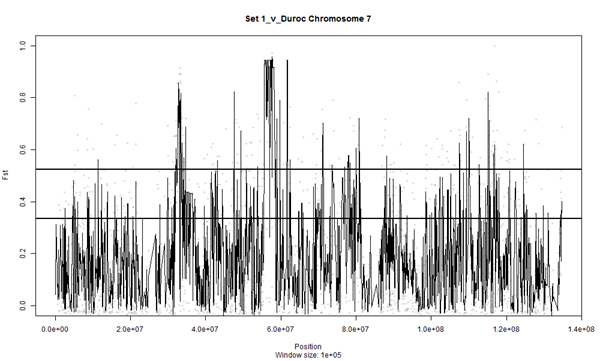

Supplement: Additional file 8: Figure S7. — FST values around the LCORL region of porcine chromosome 8. The upper panel shows population 1 against other European breeds, and the lower panel the population 2 ADPs against the same European breeds. The yellow bar represents the region 5’ to the LCORL locus, which harbours the peak SNP adjacent to this gene (*). (TIF 67 kb) [file 12864_2017_3536_MOESM8_ESM.tif]

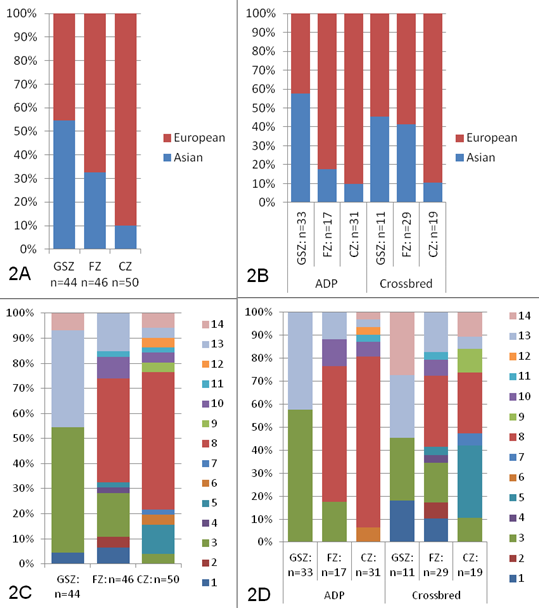

Supplement: Additional file 9: Figure S2. — Distribution of mitochondrial sequences across agro-ecological zones. Panel 2A shows the distribution of sequences according to their clustering into Asian or European clades by region: Guinea Savannah Zone (GSZ: UWR and NR); Forest Zone (FZ: ER and AR); Coastal Zone (CZ: CR and GAR). Panel 2B further subdivides the data according to local pig classification into ADP or crossbred. Panels 2C and 2D provide the same breakdown by sequence haplotype. In each panel, n = total number of animals sampled. (TIF 150 kb) [file 12864_2017_3536_MOESM9_ESM.tif]

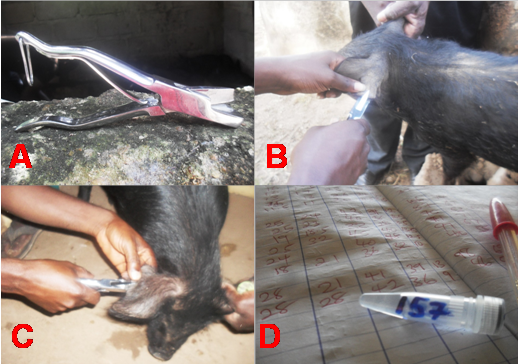

Supplement: Additional file 10: Figure S1. — Tissue sampling. Sampling porcine ear tissues using an ear notcher (A). B and C represent the procedure, the tube in D contains tissue in preservative. For each animal additional husbandry and morphological information (e.g. weight, height, length: D) was recorded on farm. (TIF 400 kb) [file 12864_2017_3536_MOESM10_ESM.tif]
