# Supplementary material for: Origin and phylogenetic status of the local Ashanti Dwarf pig (ADP) of Ghana based on genetic analysis
Source: BMC Genomics. 2017 Feb 20;18:193. doi: 10.1186/s12864-017-3536-6 (PMC5319064; doi:10.1186/s12864-017-3536-6)
Supplement: Additional file 5: — Supplementary information on sampling, methodology and analyses. (DOCX 28 kb) [file 12864_2017_3536_MOESM5_ESM.docx]

**Supplementary information**

**Sample Collection**

Animals with black coat colour and no known history of breeding with exotics were classified as local ADPs; those with spotted patterns with known crossbreeding with exotic pigs were classified as crossbreds (XBs); animals with mainly white coat colours and known commercial European genetics were classified as exotics. The exotic animals and the crossbreds were mostly from institutional and commercial farms. All animals had coat colours recorded manually, and, where possible, animals were photographed as part of the recording process. The number and type of pigs and sampled locations are as shown in Supplementary Tables: Table S1.

The coastal savannah zone is characterized by sandy beaches and marshes with land rising between 250m and 300m above sea level and an annual rainfall ranging from 1000mm along the coast to about 2000mm in the interior. It lies within latitude 5°14' north and 5°35' north and longitude 0°22 west and 0°54 west. Mean monthly temperature ranges from 24ºC in the coolest month (August) to about 30°C in the hottest months of March-April (MOFA, 2013). The forest zone lies in the equatorial climatic zone that is characterized by moderate temperatures, ranging from 22°C at nightfall to 34°C during the day. It is the wettest part of Ghana, with a bimodal rainfall pattern averaging 1, 600mm per annum (MOFA, 2013). The guinea savannah zone on the other hand lies between longitude 0° and 1° West and latitudes 10°30'N and 11°N. The natural vegetation is that of the savannah woodland characterized by short scattered drought-resistant trees and grass that gets burnt by bushfire or scorched by the sun during the long dry season. The climate is characterized by a unimodal rainfall with mean annual rainfall between 800mm and 1100mm from May/June to September/October (MOFA, 2013).

**Mitochondrial Sequence Analysis**

All sequences generated for the mitochondrial D-loop segment were screened for matches to prior GenBank entries using BLAST (100% over sequence query to subject match). The results are shown in Supplementary Tables: Table_S2. The first column gives the haplotype number as shown in figure 2 of the main manuscript. The second column gives the accession number for the matched, representative sequence deposited in the public databases. The frequency of each haplotype in the sample population is indicated as a percentage of the total sequences analysed (140 animals: column three). The majority of sequences show mismatches to prior database entries, and are therefore considered unique sequences (as shown in column four). The geographical origins of matching GenBank entries are indicated in column five.

The fewest number of mitochondrial haplotypes were observed in the Guinea Savannah zone (4 in total), and the greatest in the Coastal zone (11 in total). ADPs of the Guinea Savannah had only two mitochondrial haplotypes, one of which, sequence 3, has 100% match to AF486870 from the Dahuabai pig breed and clusters into the Asian clade. The other, sequence 13, has 100% match to JN031088, representative of mitochondrial sequences observed in Iberian pig breeds and clusters into the European clade The two additional haplotypes seen in the crossbred pigs of the Guinea Savannah zone fall into one European (sequence 14) and one Asian (sequence 1). Sequence 14 is also observed in animals from the Coastal region, and has a 100% match to a sequence observed in multiple domestic pigs from Eastern Europe and the Iberian Peninsula (e.g. JN031065), whereas sequence 1 has a match to a single entry from a domestic pig sampled in Vanuatu (DQ994636).

ADP sequences from the coastal region predominantly carry European clade member sequence 8 (unique to this study), with ADPs from the Forest zone bear evidence of all three of these major sequences from the adjacent regions (sequences 3, 8 and 13), in addition to a fourth haplotype (sequence 10: unique to this study) which is in common with ADPs from the coastal region (see Figure S2D).

Interestingly, the greatest diversity of mitochondrial sequences in crossbred local animals was also found in the intermediate, Forest, zone (see Figure S2D).

**Y chromosome Analysis**

Data from SRY sequencing showed only two *SRY* haplotypes, one of which is found in both Asian and European breeds, and one of which is found in Asian and Tamworth pigs. The former is almost exclusive in European breeds, and the latter is the only *SRY* sequence observed in Tamworth to date. Further genotyping of animals on the llumina PorcineSNP60 BeadChip confirmed extended Y chromosome haplotypes in accordance with the *SRY* data.

Supplementary Tables: Table S3 summarises the data from sequencing the *SRY* gene and Y chromosome SNP typing on the llumina PorcineSNP60 BeadChip. Animals are identified by region (AR, Ahanti; CR, Coastal; ER, Eastern; GAR, Greater Accra; UWR, Upper Western; NR, Northern), recorded gender (M, male; F, female) and collection identification number, and classified as Ashanti Dwarf Pig (ADP) or known crossbred (XB). Animal 23 is of European origin (breeds unknown) and animal 116 is Large White (LW). Animal 163 was originally defined as female, but is clearly male from the genotyping data (X chromosome homozygous: data not shown). Animal 42 failed QC overall, but appears to have a signature similar to the major Y haplotype observed across Ghana based on those SNPs which passed quality control parameters.

**Coat Colour Analysis**

Coat colour variation was observed both in local pigs classified as ADP (Figure S3A and B) and in local pigs known to have mixed genetic origins (Figure S3C and D). Across agro-ecological zones, ADPs were exclusively black-coated in the forest zone (AR and ER) whilst the majority of the variation in ADP coat colour was seen in pigs from the coastal savannah zone, particularly the GAR (Figure S4 and Supplementary Tables: Table S4). This correlates with the greater heterogeneity observed in local crossbred pigs from each agro-ecological zone, and may reflect unknown admixture in local ADP populations, and/or epistatic effects on coat colour outcome.

***MC1R* Gene Sequence Analysis**

Sequencing of the open reading frame of *MC1R*, and the putative promoter region, allowed comparison with the previously published signatures for the major haplotypes found in this gene. Data for coat colour, sequence polymorphisms and genotype predictions are summarised in Supplementary Tables: Table S4.

Analysis of the *MC1R* alleles in the sampled local pigs showed that the ADPs had both the Asian dominant black (201: 43% of black alleles) and the European dominant black (301: 57% of black alleles) variants, whilst the crossbreds also carried the European spotting variant of *MC1R* at high frequency (501: 41%) in conjunction with other European *MC1R* alleles (Figure S4). Further analysis of the distribution of *MC1R* alleles by region showed that local pigs from the Guinea Savannah zone (UWR and NR) had the highest percentage of Asian dominant black (201: 83% ADP and 100% crossbred black alleles) whilst the European dominant black sequence (301) was mainly found in the animals from the Forest zone (ER and AR: 71% ADPs and 89% crossbred black alleles) and the Coastal Savannah zone (CR and GAR: 60% ADPs and 40% crossbred black alleles) regions. The spotting allele was the major variant observed in local crossbred animals of the Guinea Savannah zone, although the total number of animals is small (64%: 9 alleles of 14 scored). This allele may also explain the occurrence of spotted offspring in the local ADP population, since 4/17 animals were heterozygous for a dominant black and a spotting variant, and one animal from a litter from the Guinea Savannah zone was homozygous for the spotting allele (animal 149).

**Analysis of Illumina PorcineSNP60 BeadChip Data**

The data from the 72 animals selected from Ghana were initially analysed against each other using a principle components analysis (PCA). “Exotic” animals are largely of commercial European breed (e.g. Large White) origin. The ADPs from the Guinea Savannah zone (UWR and NR) form a distinct cluster in the bottom left quarter whilst those from the GAR in the Coastal Savannah zone also form a distinct cluster in the lower right quarter Based on biological knowledge, PCA1 probably represents geographical distribution, and PCA2 genetic distance. At the whole genome level, ADP from Greater Accra may therefore be genetically similar to animals from the Guinea Savannah zone

The results from genotyping the 72 animals were compared independently on a global level. Subsets of SNP data were selected from the study of Burgos-Paz *et al.*, 2013. Commercial European breeds separate into two groups: Duroc (20 animals) and ‘Other’ (Large White and Landrace; 20 animals each). Chinese breeds include Meishan (17), Jinhua (16), Jiangquhai (11) and the Xiang miniature pig (13). Wild boar were selected from Hungary (1), Poland (5) and Tunisia (7). Other breeds (16 Iberian, 14 Bisaro, 4 Sicilian, and 4 Canarian Black pigs) are plotted independently. Ghanaian local pigs are categorised as ADP, crossbred or imported European commercial breeds. Here the Ghanaian pigs, regardless of classification, cluster more closely with the other European breeds.

To explore further, ADP samples from the Guinea Savannah zone were identified as “set 1” and those from the Coastal Zone were selected as “set 2”. Each subgroup of ADPs consisted of 17 animals. These were analysed against the European commercial breeds, Duroc and Chinese breeds in separate F_ST_ analyses, as outlined in Materials and Methods. The windows identified were compared, only those which were independently identified in the comparisons between ADP set 1 or ADP set 2 against the other population of pigs were taken for further scrutiny.

Intervals defined as common between each ADP set and the comparison group were analysed for gene content. Similarly, regions with high F_ST_ values between ADP set 1 and ADP set 2, which were unique to this comparison, were included in the study. All the intervals and associated genes are listed in Supplementary Tables: Table S5. Where the peak SNP fell within a gene, this locus is highlighted in bold.

Significant intervals identified regions previously reported to be important for body size in other mammals, including *LCORL*. As with other mammals, the peak falls 5’ to the start of the transcript, suggesting that the expression level of the transcript (as observed in horses: Metzger et al, 2013) may be key to explaining the effect on size.

Taking all the genes in the defined intervals, very few significant results were observed using DAVID functional annotation analysis. The most significant results are for the classifications of genes associated with disease in humans (GAD subtypes). With a cut off of p<0.05 following adjustment for multiple testing, most genes define differences in neurological function (see Supplementary Tables: Table S6): “chemical dependency” and “psychiatric” classes in humans are the most significant for the comparisons of ADPs against Duroc or European breeds, and between the two geographically defined ADP populations. Genes relating to “body weight” (ADP v Duroc) and “metabolic” traits (set 1 ADP v set 2 ADP) are also noted.

For those genes at the peaks of the F_ST_ intervals, no results were significant. However, a high percentage of loci belonged to the “cardiovascular” category (56%) in the ADP

comparison against European pigs, and “chemical dependency” category (60%) in the ADP comparison against Duroc. This latter group of genes significantly overlaps with the GAD Disease categories “metabolic” trait and “body weight” (Supplementary Tables: Table S6), suggesting that these behavioural loci potentially govern feeding behaviours as well as addiction in humans. Although not clustered in these analyses, individual gene classifications include immunological function and infection (Supplementary Tables: Table S7).

It should be noted that the genes identified also lie in intervals on the porcine QTL maps with analogous classes of phenotype: for example *SLC44A1* is associated with age of menarche in humans, and is within the QTL region for “age at puberty” in pigs. The QTL intervals tend to be large, so combining both sets of data may help to elucidate novel candidates for these traits in pigs.

These results offer potential target regions for further investigations of the genetic makeup of the local pig populations within Ghana. This will be important for future breeding programmes, both to conserve local pig breeds, and to develop robust cross bred animals with the desirable reproductive capacity and body size of many commercially important pig breeds.

**Additional bibliography**

Metzger J, Schrimpf R, Philipp U, Distl O. (2013) Expression levels of LCORL are associated with body size in horses. PLoS One. 8:e56497. doi:10.1371/journal.pone.0056497.
